# Associations between Meal Companions and Obesity in South Korean Adults

**DOI:** 10.3390/ijerph17082697

**Published:** 2020-04-14

**Authors:** Wonseok Jeong, Sung-In Jang

**Affiliations:** 1 Department of Political Science and Economics, Waseda University, Tokyo 169-8050, Japan; a63645712@gmail.com; 2 Institute of Health Services Research, Yonsei University, Seoul 03722, Korea; 3 Department of Preventive Medicine, Yonsei University College of Medicine, Seoul 03722, Korea

**Keywords:** dinner time, family dinner, meal companion, eating behavior, obesity

## Abstract

Dinner, considered the main meal of the day, forms a large portion of an individual’s overall food intake. Therefore, having family dinners has a significant impact on peoples’ health. This study examined the relationship between meal companions and obesity among South Korean adults. Data from 23,494 participants, from the 2013–2017 Korean National Health and Nutrition Examination Survey (KNHANES), were examined. Participants were divided into three categories: dinner with family, dinner with others, and dinner alone. Obesity was the dependent variable, using body mass index recommended by the KNHANES. A multinomial logistic regression analysis was performed to examine the target association. Compared to those that had family dinners, people who ate dinner with others or alone had a higher obesity risk (With Others: odds ratio (OR) = 1.19, 95% confidence interval (CI) = 1.04–1.36; Alone: OR = 1.15, 95% CI = 1.03–1.27). Participants who engaged in weekly heavy drinking were more likely to be obese than those who did not drink (OR = 1.34, 95% CI = 1.19–1.51). Moreover, those who had dinner with others or alone were at higher risk of obesity regardless of their breakfast companion. Further, people who had daily meals outside of their homes had a higher risk of obesity than those who had dinner with others and those who had family dinners. Having family dinners poses a significantly lower risk of obesity compared to having dinners with others or alone, as shown by this investigation. By detailing the correlation between meal companions and obesity, this study could help motivate dieters to have more frequent family dinners.

## 1. Introduction

The World Health Organization (WHO) states that worldwide obesity has almost tripled since 1975, meaning more than 1.9 billion adults were categorized as overweight by 2016; 38% of these adults were overweight, while 13% were categorized as obese [1]. In South Korea, the obesity rate increases every year, such that 31.8% were obesity in 2013 but that increased to 34.6% in 2018 among Korean adults [2]. Indeed, over half of adults worldwide are considered overweight when using peoples’ body mass index (BMI) as the indicator [1,3]. Obesity not only decreases a person’s quality of life, but a raised BMI can also cause serious medical complications, including various cardiovascular diseases, musculoskeletal disorders, and even some severe cancers [4,5,6]. Therefore, general awareness about obesity has aroused impassioned arguments, with copious efforts having been undertaken for more effective weight reduction strategies.

The traditional family culture has broken down in many Asian countries due to the rapidly developing nature of society. For example, the data from Statistics Korea indicates that the number of single person households in Korea was 28.6% of Korean total households in 2017, compared to 15.5% in 2000 [7]. Additionally, single person households in Korea are expected to further increase, reaching 37.3% of total households in 2047 [8]. In conjunction with these statistics, eating alone is becoming a popular new trend in Korea [9]. An increased amount of restaurants are encouraging these emerging dietary patterns by providing special seats for single customers, reflecting the fact that, no matter a person’s sex, age, or occupation, eating alone has become increasingly popularized [10]. Furthermore, the 61.8% of people in 2007 who responded that they often have dinner with their family decreased to 58.8% in 2015, showing that the tendency to engage in family dinners has decreased over time [11]. An increase in the number of working mothers, sudden economic growth, and lifestyle changes have all resulted in this reduced percentage of family dinners, consequently leading to fewer side dishes and less satisfaction experienced during family dinners [12].

Eating alone often involves unhealthy dietary intakes. People tend to eat foods without taking sufficient consideration for their nutritional quality when eating alone, and there is oftentimes a lack of healthier additions such as fruits and vegetables [13]. In addition, a previous study has found that low nutrient food consumption is highly associated with the ingestion of higher calories [14]. Notably, a dietary pattern without a sufficient amount of food variety unconsciously leads to a higher calorie intake. This led us to speculate on a potential connection between changes in dietary patterns influenced by meal companions and obesity, while utilizing BMI as the key indicator.

Various studies have already reported on the relationship between the formation of insecure mental health patterns and the presence of dinner companions [15,16]. This study, however, focuses only on the relationship between the effect of eating alone or having different companions during dinners and the occurrence of obesity. Building on this, it becomes important to investigate the association between dinner companions and peoples’ BMI in the calculation of obesity rates. 

Consequently, the purpose of this study was to examine the association between meal companions and peoples’ BMI score, while dividing meal companions into dinner with family, meal with others, and eating alone, and BMI scores into obesity, overweight, and underweight.

## 2. Methods

### 2.1. Participants

Data for this study were taken from a sample of the 2013–2017 Korean National Health and Nutrition Examination Survey (KNHANES), which is an investigation into the health of the public, the status of chronic diseases, and the status of food/nutrition. The survey was conducted by the Korea Centers for Disease Control and Prevention. The number of targeted participants for the 2013–2017 KNHANES came to 39,225. Information from individuals with missing data for the variables of interest used in this study, as well as those for individuals aged 1–18 years, were excluded. Moreover, those who did not eat both dinner and breakfast in the preceding week were also excluded. Following all exclusions, data from 23,494 participants were analyzed. 

### 2.2. Variables

The primary independent variable in this study was the individuals’ usual meal companions during the past year. Participants were classified into three groups: family dinner, dinner with others, and eating dinner alone. Individuals who answered “No” to the question “During the last year, did you usually eat dinner with others” were placed in the eating alone group. Those who reported “Yes” were asked a follow-up question, namely “During the last year, with whom did you usually eat dinner?”. Based on their answers, individuals were placed either into the “with family” or the “with people other than family” group. For the breakfast companion variables, this proceeded in the same way. Additionally, all analyses included participants’ demographic, socioeconomic, and health-related characteristics. The demographic analysis consisted of participants’ age, gender, and marital status. The socioeconomic analysis consisted of participants’ education, region, household income level, and occupation. The health-related characteristics analyzed participants’ frequencies of heavy drinking, the number of days in a week that muscular exercise was performed, self-reported health statuses, and eating out rates. 

BMI was included as the main dependent variable in this study. BMI was defined, according to the Korean guidelines, as follows: underweight: <18.5 kg/m², normal: 18.5–22.9 kg/m², overweight: 23–24.9 kg/m², and obese: ≥25 kg/m², which was also recommended by the KNHANES [17,18].

### 2.3. Statistical Analysis

A chi-square test was conducted to investigate the general characteristics of the study population. A multinomial logistic regression analysis was performed to examine the associations between dinner companions and obesity, after accounting for potential confounding variables, including demographic, socioeconomic, and health-related characteristics. Multinomial logistic regressions were used when the dependent variables contained more than two categories. Results are reported as an odds ratio (OR) with a 95% confidence interval (CI). Subgroup analyses were also performed with the multinomial logistic regression analysis to investigate the associations between breakfast companion, frequency of heavy drinking, number of days of muscular exercise, self-reported health status, and eating out rate. The analysis used a stratified sampling (k strata) and a clustering variable (primary sampling units) provided by the KNHANES. All analyses included the use of weighted variables. Differences were considered statistically significant with a *p*-value of < 0.05. All data analyses were conducted using the SAS 9.4 software (version 9.4; SAS Institute Inc., Cary, NC, USA

## 3. Results

Table 1 presents the general characteristics of the study population. Among the 23,494 participants, 7863 (33.5%) were categorized as obese, 5465 (23.3%) were overweight, 9203 (39.2%) were normal, and 963 (4.1%) were underweight according to their BMI. Having dinner with family represented 63.9% of the participants. Additionally, 11.3% of participants had dinner with others and 24.8% had dinner alone.

Table 2 shows the association between meal companions and BMI scores. Compared to people who ate dinner with family, those who had dinner with others and those who ate alone had higher risks of developing obesity. These results were statistically significant (With Others: OR = 1.19, 95% CI = 1.04–1.36; Alone: OR = 1.15, 95% CI = 1.03–1.27). Furthermore, those who had breakfast with others were at higher risk of obesity, although these results were not statistically significant. Men had a higher risk of developing obesity than women (OR = 2.18, 95% CI = 1.99–2.38). People who engaged in heavy drinking once per week had higher risks of obesity compared to those who did not drink (Obesity: OR = 1.34, 95% CI = 1.19–1.51; Overweight: OR = 1.17, 95% CI = 1.03–1.33). Higher self-reported health statuses were also related to a lower risk of obesity (High: OR = 0.64, 95% CI = 0.57–0.71; Middle: OR = 0.78, 95% CI = 0.71–0.86). These results were all statistically significant. Finally, people who reported eating out daily had a higher risk of developing obesity than those who never eat out.

Table 3 shows the results of the subgroup analyses between dinner companions and BMI scores, focusing on the breakfast companion, frequency of heavy drinking, number of days of muscular exercise, self-reported health status, and eating out rate. Those who had dinner with others or alone were at higher risk of obesity regardless of breakfast companion, compared to those who had family dinners. Due to a higher frequency of heavy drinking, those who had dinner with others or alone had higher risks of developing obesity than those who had dinners with their families. Moreover, when people engaged in heavy drinking once a week, their risk of developing obesity increased by 1.24 times compared to those who had dinner with others, and by 1.16 times for those who had dinner alone, using those who had dinner with family as the baseline. Other health-related variables, such as the number of days engaged in muscular exercise and participants’ self-reported health statuses, were associated with higher risks of obesity in those who had dinner with others or alone compared to those who had dinner with family. Notably, this risk was also higher for those who did not engage in muscle building exercises and those whose self-reported health statuses were low. Additionally, the rate of obesity development was higher in those who reported eating out daily or at least 3–6 times per week when compared to those that did not. 

## 4. Discussion

Due to the lack of authoritative information regarding obesity, people are not paying sufficient attention to the risk of becoming overweight and obese that is needed to prevent future health problems. The WHO has announced that obesity is a highly common occurrence within the world’s population, while remaining a preventable condition [1]. In this study, we detailed the connections between meal companions and people’s BMI scores through the use of demographic, socioeconomic, and health-related variables gained from the 2013–2017 KNHANES data. There was a positive association found between eating dinner alone and an increased and statistically significant risk for gaining weight. Moreover, although having family dinners is highly associated with lower risk of obesity, eating family breakfasts has no statistically significant effect. This shows that having family dinners is important.

Today, followed by the breakdown of the traditional family culture, single person households increased significantly in Korea [7,8]. As a result, eating alone has become a popular new trend that myriad restaurants even provide special seats and menus for single customers [9,10]. When having family dinner, people tend to focus more on the food that they usually consume and subsequently keep their proper amount [19]. In contrast, while having food alone, people often fail to do so and thus end up overeating [13,14]. This presents a clear and advantageous course of action for people who are trying to lose weight, in that they can aim to spend more time engaged in family dinners instead of having these meals alone. For people who do not often have chances to eat with their families, such as single person households, our study recommends to not eat while watching television or working, since distracted eating may unconsciously add the amount you eat [20]. Furthermore, having dinner with others/friends or colleagues from their workplaces too often, can lead to over drinking, which is also highly discouraged [21].

The role of the frequency of engaging in heavy drinking substantiates the notion that eating dinner with family has a negative association with one’s BMI scores. When people have dinner by themselves or with others/friends or colleagues from their workplaces, they tend to drink alcohol more heavily [21]. Alcohol consumption often occurs in social situations, during events such as company dinners or when one spends time with friends, such that during these times, the chances of people engaging in heavy drinking is high, with alcohol having been found to have a unifying effect on people and oftentimes it becomes a crucial component in social group formation [22]. Furthermore, because alcohol eases stress and increases the experience of pleasure, people will often drink, even if they are having dinner alone [23,24]. A previous study has found that drinking alone leads to an increased risk of heavier alcohol consumption and related problems, which oftentimes requires taking necessary precautions [23]. In most family dinners, however, people do not tend to drink as often as they would do in other social occasions, and even when they do, they would not engage in heavy drinking. There are 155 calories in one small can of beer, meaning that having several drinks during a social situation could add up easily to consuming a few thousands extra calories. Moreover, people usually drink alcohol containing high calories in addition to eating salty foods, which then leads to increased alcohol consumption because of the resulting thirst [21]. As a result, those who drink heavily once a week contribute significantly more to the total number of obese people than those who only drink once a month or not at all.

A lower self-reported health status reflects a heightened possibility that one is obese. When having a family dinner, an increased variety of food is more likely when compared against eating alone or with friends [13]. This is in part because the more people there are during dinner, the more likely they are to serve more diverse food and, when having a family dinner, people are more cautious toward promoting the family’s health. Indeed, family meals play an important role in promoting positive dietary intakes. One study discovered that family meals tend to provide people with a wider variety of nutrients, including protein, calcium, vitamins, and fiber, which are all essential to peoples’ health [25]. More frequently eating balanced foods, in terms of their nutrients, leads one to be more confident toward their health status and, therefore, people having frequent family dinners are more likely to check “high” on self-reported health status surveys. In summation, people who often have family members as their meal companions tend to report higher self-reported health statuses. 

According to a study on the consumption habits of adults, when eating out, people tend to eat greater amounts of calories compared to when they eat dinner at home [26]. Dishes from restaurants are mostly high in fats and low in nutrients when compared to food cooked at home, as these businesses oftentimes lend higher priority on taste and convenience rather than nutrition and health. A previous study has found that people who tend to eat out received at least 25% of their daily energy intake through the restaurant meal [27]. Meanwhile, as the Korean food delivery system is rising in popularity, people living in single person households receive most of their dinners from this system in order to save the time and effort of preparing a meal themselves [28]. In addition to this, unlike eating out with others or eating alone, people are more likely to prepare their own food when having dinner with family. Consequently, people who eat with others or eat alone tend to eat dinner outside of the home, ending up with higher calorie intakes. 

Our study does have several limitations. First, the results of this study are based on self-report measures, especially for the health status measurements. Thus, some survey questions might be subject to recall bias. Therefore, caution should be taken when interpreting these results. Second, due to this study’s cross-sectional design, cause and effect, as well as the direction of the relationships observed, could not be determined. Third, we could not take into consideration the difference between the effects of weekend and weekdays, due to a lack of appropriate data. Fourth, as many people usually had breakfast with family or alone, there was lack of individuals who had breakfast with others. Despite their low numbers, weight variables developed by KNHANES improved the representativeness of the sample. Finally, some studies considered waist circumference or waist-to-hip ratio as indicators of obesity [29,30]. Indeed, BMI might not always be an accurate obese indicator. For instance, it is not appropriate to label the people who said they work out five times a week as obese, just because their BMI scores are high [31]. The people who regularly do muscular exercises are less likely obese even though their height to weight ratios are pretty high due to their high muscle masses, as BMI does not regard the body fat percentages nor muscle mass in its calculation [31]. Nonetheless, BMI is commonly used as a reliable predictor of obesity, as it takes into consideration other increased levels of health risk factors for all ages [1,18].

Despite these limitations, our study does possess several strengths. The KNHANES is conducted by a national institution based on a random cluster sampling and, therefore, the data gained from it is statistically reliable and representative compared to surveys performed by private institutions. Moreover, as this study was conducted over five consecutive years using weight variables developed by the KNHANES, the representativeness of the sample was improved upon. Furthermore, KNHANES data is derived from health interviews, which included both physical examinations and nutrition surveys, that form a reliable base for the creation of health-related policies and programs [32]. This study can therefore be the baseline for motivating dieters to have more frequent family dinners. 

## 5. Conclusions

The general public awareness regarding BMI has shifted substantially from the impact of malnutrition to that of obesity and being overweight, as proven by recent investigations of the WHO [33]. As obesity predisposes a person to other severe diseases, it is important for a person to maintain a weight in a safe range [4,5,6]. Prior studies have proven that losing weight results in a multitude of health benefits, such as a decreased risk of diabetes, lowered blood pressure, and improved cholesterol levels [34,35]. Our research identified the relationship between meal companions and BMI scores. We discovered that people who had dinner either alone or with others were more likely to be obese than those who had dinners with their family. Understanding the various factors that lead to over consumption could greatly reduce obesity and all related diseases. The results of this study could be used to motivate people struggling with obesity to have dinner with their family through its detailing and discovery of the association with one’s meal companions and their overall BMI score.

## Figures and Tables

**Table 1:** General characteristics of study population.

Variables	Total		Body Mass Index								
			Obesity		Overweight		Normal		Underweight		*p*-Value
	*n*	(%)	*n*	(%)	*n*	(%)	*n*	(%)	*n*	(%)	
**Total**	23,494	(100.0)	7863	(33.5)	5465	(23.3)	9203	(39.2)	963	(4.1)	
**Dinner Companion**											<0.0001
With Family	15,013	(63.9)	4856	(32.3)	3509	(23.4)	6011	(40.0)	637	(4.2)	
With Others	2660	(11.3)	948	(35.6)	590	(22.2)	999	(37.6)	123	(4.6)	
Alone	5821	(24.8)	2059	(35.4)	1366	(23.5)	2193	(37.7)	203	(3.5)	
**Breakfast Companion**											<0.0001
With Family	10,748	(45.7)	3612	(33.6)	2669	(24.8)	4099	(38.1)	368	(3.4)	
With Others	647	(2.8)	273	(42.2)	139	(21.5)	218	(33.7)	17	(2.6)	
Alone	12,099	(51.5)	3978	(32.9)	2657	(22.0)	4886	(40.4)	578	(4.8)	
**Gender**											<0.0001
Male	9647	(41.1)	3722	(38.6)	2517	(26.1)	3134	(32.5)	274	(2.8)	
Female	13,847	(58.9)	4141	(29.9)	2948	(21.3)	6069	(43.8)	689	(5.0)	
**Age (year)**											<0.0001
19–29	2704	(11.5)	661	(24.4)	433	(16.0)	1332	(49.3)	278	(10.3)	
30–39	3799	(16.2)	1146	(30.2)	707	(18.6)	1725	(45.4)	221	(5.8)	
40–49	4246	(18.1)	1376	(32.4)	999	(23.5)	1738	(40.9)	133	(3.1)	
50–59	4460	(19.0)	1630	(36.5)	1151	(25.8)	1583	(35.5)	96	(2.2)	
≥60	8285	(35.3)	3050	(36.8)	2175	(26.3)	2825	(34.1)	235	(2.8)	
**Education Level**											<0.0001
Middle school or less	7501	(31.9)	2958	(39.4)	1940	(25.9)	2390	(31.9)	213	(2.8)	
High school	6362	(27.1)	2155	(33.9)	1482	(23.3)	2516	(39.5)	209	(3.3)	
College or over	9631	(41.0)	2750	(28.6)	2043	(21.2)	4297	(44.6)	541	(5.6)	
**Marital Status**											<0.0001
Married	16,611	(70.7)	5633	(33.9)	4,046	(24.4)	6410	(38.6)	522	(3.1)	
Separated or divorced	3372	(14.4)	1275	(37.8)	832	(24.7)	1150	(34.1)	115	(3.4)	
Unmarried	3511	(14.9)	955	(27.2)	587	(16.7)	1643	(46.8)	326	(9.3)	
**Region**											<0.0001
Urban	10,699	(45.5)	3374	(31.5)	2509	(23.5)	4364	(40.8)	452	(4.2)	
Rural	12,795	(54.5)	4489	(35.1)	2956	(23.1)	4839	(37.8)	511	(4.0)	
**Household Income Level**											<0.0001
Quartile 1 (lowest)	4639	(19.7)	1704	(36.7)	1118	(24.1)	1625	(35.0)	192	(4.1)	
Quartile 2	5892	(25.1)	2088	(35.4)	1369	(23.2)	2199	(37.3)	236	(4.0)	
Quartile 3	6357	(27.1)	2131	(33.5)	1409	(22.2)	2570	(40.4)	247	(3.9)	
Quartile 4 (highest)	6606	(28.1)	1940	(29.4)	1569	(23.8)	2809	(42.5)	288	(4.4)	
**Frequency of Heavy Drinking**											<0.0001
None	13,843	(58.9)	4426	(32.0)	3175	(22.9)	5628	(40.7)	614	(4.4)	
Once a month	5826	(24.8)	1869	(32.1)	1351	(23.2)	2352	(40.4)	254	(4.4)	
Once a week	3825	(16.3)	1568	(41.0)	939	(24.5)	1223	(32.0)	95	(2.5)	
**Occupational Classification**											<0.0001
White-collar	5278	(22.5)	1572	(29.8)	1197	(22.7)	2262	(42.9)	247	(4.7)	
Blue-collar	5474	(23.3)	2094	(38.3)	1367	(25.0)	1885	(34.4)	128	(2.3)	
Pink-collar	2856	(12.2)	992	(34.7)	672	(23.5)	1080	(37.8)	112	(3.9)	
None	9886	(42.1)	3205	(32.4)	2229	(22.5)	3976	(40.2)	476	(4.8)	
**Number of Days of Muscular Exercise**											<0.0001
None	17,978	(76.5)	6080	(33.8)	4094	(22.8)	7019	(39.0)	785	(4.4)	
1–2	2094	(8.9)	689	(32.9)	487	(23.3)	832	(39.7)	86	(4.1)	
3–4	1730	(7.4)	532	(30.8)	429	(24.8)	719	(41.6)	50	(2.9)	
≥5	1692	(7.2)	562	(33.2)	455	(26.9)	633	(37.4)	42	(2.5)	
**Self-Reported Health Status**											<0.0001
High	6848	(29.1)	2058	(30.1)	1628	(23.8)	2924	(42.7)	238	(3.5)	
Middle	11,975	(51.0)	3972	(33.2)	2858	(23.9)	4674	(39.0)	471	(3.9)	
Low	4671	(19.9)	1833	(39.2)	979	(21.0)	1605	(34.4)	254	(5.4)	
**Eating Out Rate**											<0.0001
Everyday	5094	(21.7)	1776	(34.9)	1252	(24.6)	1857	(36.5)	209	(4.1)	
3–6 times per week	11,391	(48.5)	3602	(31.6)	2578	(22.6)	4732	(41.5)	479	(4.2)	
None	7009	(29.8)	2485	(35.5)	1635	(23.3)	2614	(37.3)	275	(3.9)	
**Year**											0.0124
2013	4657	(19.8)	1494	(32.1)	1088	(23.4)	1866	(40.1)	209	(4.5)	
2014	4405	(18.7)	1389	(31.5)	1025	(23.3)	1801	(40.9)	190	(4.3)	
2015	4447	(18.9)	1520	(34.2)	1068	(24.0)	1685	(37.9)	174	(3.9)	
2016	4915	(20.9)	1721	(35.0)	1113	(22.6)	1886	(38.4)	195	(4.0)	
2017	5070	(21.6)	1739	(34.3)	1171	(23.1)	1965	(38.8)	195	(3.8)	

**Table 2:** Factors associated with body mass index.

Variables	Obesity		Overweight		Underweight	
	Adjusted OR	95% CI	Adjusted OR	95% CI	Adjusted OR	95% CI
**Dinner Companion**						
With Family	1.00		1.00		1.00	
With Others	1.19 *	(1.04–1.36)	1.03	(0.89–1.19)	0.86	(0.65–1.14)
Alone	1.15 *	(1.03–1.27)	1.11	(0.99–1.23)	0.68 *	(0.53–0.86)
**Breakfast Companion**						
With Family	1.00		1.00		1.00	
With Others	1.16	(0.91–1.46)	0.81	(0.62–1.06)	0.92	(0.52–1.63)
Alone	0.96	(0.89–1.04)	0.91	(0.83–1.00)	1.23 *	(1.02–1.48)
**Gender**						
Male	2.18 *	(1.99–2.38)	1.83 *	(1.65–2.02)	0.75 *	(0.61–0.93)
Female	1.00		1.00		1.00	
**Age (year)**						
19–29	0.82	(0.67–1.01)	0.63 *	(0.50–0.80)	2.18 *	(1.49–3.17)
30–39	1.02	(0.88–1.19)	0.77 *	(0.66–0.91)	1.74 *	(1.27–2.37)
40–49	1.11	(0.97–1.28)	0.92	(0.80–1.06)	1.11	(0.83–1.49)
50–59	1.16 *	(1.04–1.31)	1.05	(0.92–1.19)	0.94	(0.70–1.25)
≥60	1.00		1.00		1.00	
**Education Level**						
Middle school or less	1.77 *	(1.55–2.02)	1.64 *	(1.41–1.90)	0.92	(0.65–1.31)
High school	1.14 *	(1.04–1.26)	1.16 *	(1.04–1.29)	1.00	(0.77–1.28)
College or over	1.00		1.00		1.00	
**Marital Status**						
Married	1.25 *	(1.06–1.47)	1.29 *	(1.08–1.54)	0.57 *	(0.44–0.76)
Separated or divorced	1.35 *	(1.10–1.65)	1.40 *	(1.13–1.74)	0.63 *	(0.44–0.91)
Unmarried	1.00		1.00		1.00	
**Region**						
Urban	0.90 *	(0.83–0.97)	0.98	(0.91–1.07)	1.03	(0.88–1.21)
Rural	1.00		1.00		1.00	
**Household Income Level**						
Quartile 1 (lowest)	1.10	(0.95–1.26)	1.00	(0.86–1.15)	1.13	(0.86–1.50)
Quartile 2	1.13 *	(1.02–1.26)	1.00	(0.89–1.13)	1.09	(0.87–1.37)
Quartile 3	1.11 *	(1.00–1.23)	0.96	(0.86–1.07)	0.90	(0.73–1.11)
Quartile 4 (highest)	1.00		1.00		1.00	
**Frequency of Heavy Drinking**						
None	1.00		1.00		1.00	
Once a month	1.09	(0.98–1.20)	1.09	(0.98–1.21)	0.87	(0.72–1.05)
Once a week	1.34 *	(1.19–1.51)	1.17 *	(1.03–1.33)	0.64 *	(0.49–0.85)
**Occupational Classification**						
White-collar	0.98	(0.88–1.09)	1.07	(0.94–1.20)	0.94	(0.75–1.17)
Blue-collar	0.97	(0.87–1.07)	0.95	(0.84–1.07)	0.79	(0.61–1.02)
Pink-collar	1.10	(0.97–1.24)	1.13	(0.98–1.29)	1.04	(0.80–1.35)
None	1.00		1.00		1.00	
**Number of Days of Muscular Exercise**						
None	1.15	(0.99–1.34)	0.93	(0.78–1.09)	1.69 *	(1.14–2.51)
1–2	1.14	(0.94–1.38)	0.98	(0.79–1.20)	1.40	(0.89–2.19)
3–4	1.02	(0.84–1.23)	0.97	(0.80–1.19)	1.04	(0.63–1.72)
≥5	1.00		1.00		1.00	
**Self-Reported Health Status**						
High	0.64 *	(0.57–0.71)	1.02	(0.90–1.15)	0.41 *	(0.33–0.52)
Middle	0.78 *	(0.71–0.86)	1.06	(0.95–1.19)	0.56 *	(0.45–0.68)
Low	1.00		1.00		1.00	
**Eating Out Rate**						
Everyday	1.07	(0.94–1.22)	1.33 *	(1.16–1.54)	0.95	(0.73–1.25)
3–6 times per week	1.04	(0.95–1.15)	1.09	(0.98–1.21)	0.87	(0.71–1.08)
None	1.00		1.00		1.00	
**Year**						
2013	0.87 *	(0.78–0.98)	0.94	(0.83–1.06)	1.1.5	(0.89–1.48)
2014	0.86 *	(0.76–0.98)	0.93	(0.82–1.05)	0.98	(0.77–1.26)
2015	0.99	(0.88–1.11)	1.03	(0.90–1.16)	1.06	(0.82–1.36)
2016	1.08	(0.96–1.21)	1.01	(0.89–1.15)	1.03	(0.81–1.31)
2017	1.00		1.00		1.00	

Statistically significant was marked as *. Abbreviations: OR = Odds Ratios, CI = confidence intervals.

**Table 3:** Subgroup analysis associations between dinner companions and body mass index stratified by covariates.

Variables	Obesity					Overweight					Underweight				
	Dinner Companion					Dinner Companion					Dinner Companion				
	With Family	With Others		Alone		With Family	With Others		Alone		With Family	With Others		Alone	
	OR	OR	95% CI	OR	95% CI	OR	OR	95% CI	OR	95% CI	OR	OR	95% CI	OR	95% CI
**Breakfast Companion**															
With Family	1.00	1.23	(0.97–1.57)	1.38 *	(1.14–1.67)	1.00	0.89	(0.68–1.18)	1.08	(0.88–1.32)	1.00	1.11	(0.65–1.87)	0.67	(0.39–1.16)
With Others	1.00	1.40	(0.87–2.26)	1.04	(0.53–2.04)	1.00	1.05	(0.58–1.89)	0.58	(0.24–1.41)	1.00	0.87	(0.29–2.59)	0.08	(0.00–1.58)
Alone	1.00	1.13	(0.96–1.34)	1.03	(0.91–1.17)	1.00	1.10	(0.92–1.32)	1.15 *	(1.00–1.32)	1.00	0.80	(0.56–1.13)	0.68 *	(0.51–0.90)
**Frequency of Heavy Drinking**															
None	1.00	1.14	(0.92–1.42)	1.10	(0.97–1.24)	1.00	0.99	(0.78–1.27)	1.07	(0.93–1.24)	1.00	0.88	(0.59–1.30)	0.76	(0.57–1.00)
Once a month	1.00	1.18	(0.94–1.47)	1.21	(0.98–1.49)	1.00	1.11	(0.87–1.41)	1.18	(0.97–1.45)	1.00	0.70	(0.44–1.11)	0.42 *	(0.25–0.69)
Once a week	1.00	1.24	(0.95–1.62)	1.16	(0.90–1.49)	1.00	0.98	(0.72–1.32)	1.15	(0.86–1.53)	1.00	1.64	(0.85–3.16)	1.18	(0.59–2.36)
**Number of Days of Muscular Exercise**															
None	1.00	1.18 *	(1.01–1.37)	1.07	(0.95–1.20)	1.00	0.97	(0.81–1.15)	1.12 *	(1.00–1.27)	1.00	0.95	(0.71–1.29)	0.68 *	(0.52–0.87)
1–2	1.00	1.08	(0.74–1.59)	1.58 *	(1.13–2.20)	1.00	1.13	(0.73–1.74)	1.17	(0.81–1.70)	1.00	0.68	(0.31–1.48)	0.91	(0.46–1.78)
3–4	1.00	0.97	(0.63–1.50)	0.88	(0.61–1.27)	1.00	1.10	(0.71–1.71)	0.89	(0.59–1.35)	1.00	0.48	(0.15–1.47)	0.40	(0.13–1.23)
≥5	1.00	1.68 *	(1.02–2.77)	2.20 *	(1.50–3.22)	1.00	1.11	(0.64–1.90)	1.17	(0.77–1.78)	1.00	0.21	(0.03–1.71)	0.67	(0.22–2.00)
**Self-Reported Health Status**															
High	1.00	1.02	(0.81–1.29)	1.28 *	(1.04–1.57)	1.00	1.00	(0.79–1.28)	1.17	(0.95–1.45)	1.00	0.72	(0.45–1.15)	0.51 *	(0.30–0.87)
Middle	1.00	1.33 *	(1.11–1.60)	1.18 *	(1.03–1.35)	1.00	1.08	(0.89–1.33)	1.18 *	(1.02–1.36)	1.00	0.92	(0.62–1.38)	0.70 *	(0.50–0.97)
Low	1.00	1.13	(0.78–1.65)	0.89	(0.71–1.12)	1.00	0.89	(0.56–1.43)	0.83	(0.63–1.08)	1.00	1.14	(0.60–2.15)	0.73	(0.47-1.14)
**Eating Out Rate**															
Everyday	1.00	1.14	(0.95–1.37)	1.09	(0.86–1.37)	1.00	0.98	(0.79–1.20)	1.04	(0.81–1.34)	1.00	0.78	(0.52–1.17)	0.54 *	(0.32–0.92)
3–6 times per week	1.00	1.26 *	(1.01–1.58)	1.21 *	(1.04–1.40)	1.00	1.04	(0.81–1.34)	1.12	(0.96–1.30)	1.00	0.98	(0.62–1.54)	0.87	(0.64–1.20)
None	1.00	0.89	(0.56–1.42)	1.03	(0.85–1.24)	1.00	1.31	(0.76–2.24)	1.12	(0.91–1.38)	1.00	0.90	(0.30–2.69)	0.49 *	(0.33–0.72)

Statistically significant was marked as *. Abbreviations: OR = Odds Ratios, CI = confidence intervals.
